# Experiences of ageism and digital technology use among older adults

**DOI:** 10.3389/fpsyg.2025.1669321

**Published:** 2025-11-21

**Authors:** Gabriele Gudynaite, Olga Zamalijeva, Vilmante Pakalniskiene, Goda Gegieckaite

**Affiliations:** Institute of Psychology, Vilnius University, Vilnius, Lithuania

**Keywords:** ageism, digital technology, older adults, digital divide, qualitative research, focus groups, internalized ageism, relational ageism

## Abstract

**Objective:**

Digital inclusion offers many opportunities to support well-being. However, the digital divide among older adults remains a significant barrier. While various technology-related factors have been identified, the impact of ageism on older adults’ use of digital technologies remains under-researched. This study aimed to analyze how older adults’ experiences of ageism relate to their use of digital technologies. It focused on internalized and relational ageism and explored confident and hesitant users’ experiences of ageism in relation to digital technology use.

**Methods:**

The study employed a qualitative research strategy using focus group discussions. Thirteen older adults (4 males, 9 females), aged 65–82, participated in the study. Two focus group discussions were organized: the first consisted mostly of confident digital technology users (6 participants), and the second of mostly hesitant users (7 participants). Thematic analysis was employed, and the data were analyzed using ATLAS.ti (version 8).

**Results:**

Internalized and relational ageism experiences were related to participants’ use of digital technologies. The potential multifaceted influence of internalized ageism on digital technology use was observed. Participants reported both subtle and overt forms of relational ageism, which related to their digital engagement in similarly negative ways. A potential interaction between relational and internalized ageism regarding participants’ use of digital technologies was observed. Confident and hesitant users differed in their experiences and responses to ageism. Confident users described social interactions as helping them resist ageist views, while hesitant users recalled interactions that reinforced stereotypes. Unexpectedly, a possible paradoxical impact of experiences of ageism was observed: for confident participants, it motivated digital technology use, whereas for hesitant users, it hindered it.

**Conclusion:**

The study demonstrated that ageism shapes older adults’ experiences with digital technologies and should be considered in future research on the digital divide. Further studies should replicate findings in other socio-cultural contexts, explore individual differences that explain why some older adults may be resilient to ageism while others more vulnerable. Additionally, research should continue to explore the broader impact of ageism across different areas of older adults’ lives.

## 1 Introduction

The use of digital technologies such as smartphones, computers, and tablets have become interchangeable part of our everyday life (Smits et al., 2022), offering numerous resources and opportunities to support well-being (Amundsen, 2021; UNECE, 2021). However, the digital divide among older adults remains a significant barrier preventing them from fully benefiting from the digital revolution (Mubarak and Suomi, 2022). Older age, defined in many European countries as 65 and above based on typical retirement age and age-policy conventions (Eurostat, 2020), has been extensively researched as a barrier to technology use (Álvarez-García et al., 2019; Zhang, 2023). Many factors influencing older people’s adoption and use of technology have been studied and identified (McDonough, 2016; Astell et al., 2020), with models specifically designed for older adults (Chen and Chan, 2014). However, existing models rarely include factors like ageism, despite evidence showing its impact even in studies where it is not explicitly investigated (Barrie et al., 2021; McCashin et al., 2022). Therefore, this field requires further development.

While age refers to a person’s chronological years, ageism refers to stereotypes, prejudice, and discrimination directed against individuals based on their age (Butler, 1969; Ayalon and Tesch-Römer, 2018). Ageism reflects societal bias and is distinct from age as a demographic factor or from psychological reactions to ageism, such as technology anxiety, which may mediate the relationship between ageism and digital technology use (Chen and Chan, 2014; Jarvis et al., 2020). This phenomenon is deeply embedded in our society and has a significant impact on older adults, as it remains one of the most normalized and least recognized forms of discrimination (Gendron et al., 2018; Morrow-Howell et al., 2020). Its impact is understood as multidimensional, global, far-reaching (Chang et al., 2020; WHO, 2021), and extending to the field of digital technology use. For a long time, older people have been portrayed as less capable and less willing to learn technological skills, with a decreased openness to change (Ivan and Cutler, 2021). Despite studies disproving this view (Mitzner et al., 2010; Vaportzis et al., 2017; van Kampen et al., 2023), society at large, as well as older adults themselves, often continue to believe such ageist stereotypes (Mannheim et al., 2021, 2023b).

The ways in which ageist stereotypes impact digital technology use can be analyzed using the Risk of Ageism Model (Swift et al., 2017), which identifies three distinct mechanisms. Stereotype embodiment explains how stereotypes about aging become internalized, shaping individuals’ expectations of their own aging and exerting influence through embodiment – conforming to stereotypical beliefs and behaviors (Levy, 2009; Swift et al., 2017). In the context of digital technology use, this can manifest as low self-efficacy, stereotypical beliefs, and doubts about one’s own digital capabilities (Mannheim et al., 2023b). Stereotype threat refers to the anxiety in situations where an individual risks confirming a stereotype (Steele and Aronson, 1995), which can cause stress, lower engagement, and impact performance (Mariano et al., 2020; Ivan and Cutler, 2021). Age discrimination impacts individuals directly through access to services and resources, or indirectly through the experience and consequences of stress (Lyons et al., 2018). In the technology domain, this has been associated with lower use rates and reduced effectiveness of technology training programs (McCausland et al., 2015; Choi et al., 2020). Despite the relevance of existing models explaining digital technology use, there is a lack of models specifically created or adapted to explain the interaction of aging, ageism, and digital technology use (Mannheim and Köttl, 2024). This gap may contribute to methodological limitations and restricted theoretical approaches, resulting in incomplete and inconsistent findings in research on the intersection of ageism and digital technology use.

Ageism is said to operate on multiple levels, including institutional, interpersonal, and intrapersonal, and can manifest both implicitly and explicitly (Levy, 2001, 2009; Ishikawa, 2023). Over the years, various forms of ageism have been identified and studied, including positive ageism, benevolent ageism, internalized ageism, relational ageism and everyday ageism (Minichiello et al., 2000; Levy, 2009; Gendron et al., 2018; Vale et al., 2020; Allen et al., 2022). Relational ageism refers to ageism that manifests in social interactions, where ageist assumptions or behaviors are expressed and reinforced (Gendron et al., 2018). It may appear in overt forms, such as being insulted or treated poorly, as well as in more subtle forms, such as being ignored or patronized (Abrams et al., 2011). Internalized ageism occurs when individuals direct ageist stereotypes toward themselves, which can negatively affect their self-perceptions of aging (Levy, 2001; Ishikawa, 2023). In relation to older adults’ use of technology, some argue that research has primarily focused on overt, discriminatory practices, particularly in the employment sector, while more subtle and internalized forms of ageism have been overlooked (Schreurs et al., 2017; Barrie et al., 2021; Chasteen et al., 2022). However, some studies suggest that age discrimination has not received sufficient attention in the field of information and communication technologies (Köttl et al., 2023). Older adults’ interactions with technology depend both on how they are perceived by society and how they perceive themselves (Ivan and Cutler, 2021). Nevertheless, there is a lack of research that integrates societal, relational, and internalized forms of ageism (Allen et al., 2022).

The impact of ageism on older adults’ use of digital technology also remains insufficiently understood. While some studies show that ageism impacts older people’s use of digital technologies (Barrie et al., 2021; Köttl et al., 2023; Mannheim et al., 2023a), others contradict this or emphasize the importance of additional factors shaping this relationship (Choi et al., 2020; Rampioni et al., 2021; van Kampen et al., 2023). Ageism manifests in diverse ways across different contexts, with significant variation in how it is experienced, expressed, and interpreted (Keskinen et al., 2023). There are many factors that may influence, mediate, or moderate its impact (Fowler et al., 2015; Kim et al., 2016; McDonough, 2016). Another important, yet underrepresented, factor that may shape experiences of ageism is the consideration of different digital user types. Research suggests that the difference between younger and older technology users lies not in actual use, but in confidence with technology (Mitzner et al., 2010). Confidence is relevant beyond access, as it can enhance older adults’ satisfaction with digital transformation (Baek, 2025). Frequency of technology use and self-rated digital skills are important indicators associated with differing levels of confidence (Scanlon et al., 2015; Schwarz, 2024). Based on similar categorizations of “open” and “reluctant” technology users, which are associated with older adults’ confidence levels (Lin et al., 2025), older digital technology users can be classified as confident or hesitant users according to their average technology use and self-rated skills (Scanlon et al., 2015).

Based on this, a qualitative research approach that allows researchers to study the experiences of ageism in relation to digital technology use might be useful. Research on ageism could also be expanded by including multiple forms of ageism and examining different types of technology users. Thus, the aim of this study was to analyze how older adults’ experiences of ageism relate to their use of digital technologies. The focus was on internalized and relational ageism, and on how confident and hesitant digital technology users experience ageism. The research objectives were:

1. To explore experiences of internalized ageism in relation to digital technology use;
2. To explore experiences of relational ageism in relation to digital technology use;
3. To examine the experiences of ageism of confident and hesitant digital technology users.

## 2 Materials and methods

### 2.1 Study design and participants

The study employed a qualitative research strategy using focus group discussions, in which a small group of individuals (from 6 to 8 people) participates in a structured discussion on the research topic led by a group moderator (Morgan, 1997). This method was selected as it facilitated the collection of rich, in-depth information from a diverse group of participants within a limited timeframe. Additionally, survey data were gathered on the basic demographic characteristics of the participants, and their digital technology skills and use. The study was conducted in compliance with ethical standards and received institutional approval by the Committee on Research Ethics in Psychology at Vilnius University.

A total of 13 community-dwelling older adults participated in the study. The focus groups were conducted at two local senior centers. The demographic characteristics of the participants are shown in Table 1. Based on the assumption that confidence in digital technology use may be particularly important for older adults (Mitzner et al., 2010; Baek, 2025), and that it can be assessed by examining participants’ frequency of use and self-rated digital skills (Scanlon et al., 2015; Schwarz, 2024), we chose to measure confidence by inquiring about participants’ frequency of use and self-rated digital skills. Six participants rated their digital technology skills as average or above, used digital technologies at least four times per week, and were defined as confident users. Seven participants rated their digital technology skills as below average, used digital technologies less than three times per week, and were defined as hesitant users. The original plan was to form one group consisting of confident digital technology users and another of hesitant users. However, due to scheduling issues that prevented some participants from attending their designated groups, the final composition resulted in one group consisting mostly of confident digital technology users and the other mostly of hesitant users. Despite this, given the high dominance of group characteristics (only one participant in each group did not align with the majority) and the approach of analyzing each participant’s narratives separately, we decided to consider this grouping as a framework that still allowed us to examine important experiences of different types of users. There were four male and nine female participants, aged 65–82 years. Most participants were retired (10 out of 13). Five of the participants had a university education, three had college education, three had post-secondary non-tertiary education, and two had upper secondary education. Most participants (8 out of 13) lived with a partner, one had a partner living outside the household, and four did not have a partner.

**TABLE 1:** Demographic characteristics of study participants.

Name	Gender	Age	Employment	Education	Partnership situation	Confidence level
Dominic	Male	79	Retired	University education	Partner in household	Confident user
Laura	Female	81	Retired	Post-secondary non-tertiary education	Partner in household	Confident user
Mary	Female	80	Retired	University education	No partner in household	Confident user
Monica	Female	70	Not employed	University education	Partner outside household	Confident user
Richard	Male	74	Retired	University education	Partner in household	Confident user
Violet	Female	65	Employed	College education	Partner in household	Confident user
Anna	Female	68	Employed	College education	Partner in household	Hesitant user
Elena	Female	82	Retired	Upper secondary	Partner in household	Hesitant user
Evelyn	Female	81	Retired	University education	Partner in household	Hesitant user
John	Male	79	Retired	Post-secondary non-tertiary education	No partner in household	Hesitant user
Natalie	Female	82	Retired	College education	No partner in household	Hesitant user
Tom	Male	82	Retired	Upper secondary	Partner in household	Hesitant user
Valerie	Female	75	Retired	Post-secondary non-tertiary education	No partner in household	Hesitant user

### 2.2 Procedure

Two months before the focus groups, informative lectures were held at local senior centers to present the research and recruit participants. Information was also shared via social media, community centers, and libraries. Interested individuals contacted the researcher, provided basic information (e.g., technological skills and frequency of use), and received details about informed consent. They were assigned to one of two focus groups, which met in September and October 2024 at two local community centers. Participants were primarily recruited from senior activity centers and social media, which tend to attract more active and socially engaged individuals. However, this might have influenced recruitment bias as it excludes less socially active or digitally disengaged older adults.

At the start of each session, the moderator introduced herself, explained the study and consent process, obtained signed consent, and collected demographic data (such as age, gender, employment and relationship status, level of education). Before the discussion, the moderator outlined rules on respect, confidentiality, and valuing all perspectives. Participants were encouraged to share their views, while the moderator ensured balanced participation, addressed sensitive issues, and intervened to prevent conflict or dominance.

The discussion began with an introductory question presenting the term “ageism” and asking participants about their experiences: “Sometimes, based on a person’s age, they are unfairly treated by others, or in a stereotypical way (e.g., it’s assumed that a person cannot hold certain job positions or acquire certain skills because of their age). This kind of experience is called ageism. Have you ever had such an experience? If so, in what situations, and how did it manifest?” Although this could have created a priming effect, during our informative lectures it was observed that potential participants often understood ageism narrowly as age-based discrimination, overlooking its broader manifestations. We therefore included a broader definition, consistent with other qualitative research (Carlasare et al., 2025). Before the discussion, participants were also encouraged to describe experiences in their own words and were reminded that there were no right or wrong answers. This approach helped balance the provided definition with open exploration.

The second question was: “How do you perceive yourself as a user of digital technologies?” This was followed by a third question: “How do you think your age impacts your use of digital technologies?” After that, participants were asked: “How do others (e.g., relatives, strangers) perceive you as a user of digital technologies?” and the final question was: “To what extent, and in what ways do such perceptions of others affect your use of digital technologies?” The discussions were audio-recorded for later transcription. Both discussions lasted approximately 1.5 h.

### 2.3 Data analysis

During the transcription process, data concerning participants’ personal information was removed, and the participants’ names were changed. A six-step thematic analysis framework (Braun and Clarke, 2006) was applied to analyze the discussion transcripts using an inductive approach to theme identification. The inductive process allowed for open coding, enabling the identification of patterns, meanings, and ideas that emerged naturally from the participants’ narratives. Inductive codes were first developed from participants’ narratives, without reference to any theoretical framework. The coding process was conducted iteratively and collaboratively, with codes emerging directly from the data to ensure that participants’ subjective experiences were represented. No codes were altered or merged to force a theoretical fit. Data were analyzed using ATLAS.ti (version 8), which facilitated coding and theme identification.

The analysis followed a systematic thematic approach, which began with data familiarization through repeated reading of transcripts. Initial codes were developed inductively from both explicit and underlying meanings in the data. These codes were then applied across the dataset and organized into subthemes and broader themes. The primary researcher developed the initial coding system. Transcripts and initial codes were then shared with two additional researchers experienced in research with older adults. Through collaborative discussions, the initial codes and manuscript content were reviewed, and the coding structure was refined based on these discussions. In cases where consensus about codes could not be reached, the team discussed differing perspectives until an agreement was reached on the most appropriate coding. Thematic saturation was reached when constant comparison showed no new codes or themes emerging and subsequent data fit existing categories. In the end, the final version of themes, subthemes and extracts from data illustrating them were reviewed with one additional researcher experienced in qualitative research field and the formulation of codes as well as their appropriateness for data was adjusted during group discussions.

Final themes reflected the interpretation of subthemes in relation to the research questions. For the first two objectives, themes and subthemes common to all research participants were analyzed and selected. For the third objective, only themes and subthemes that were common or expressed differently between hesitant and confident users were analyzed and selected. Finally, a detailed analytical narrative was produced, integrating data extracts with interpretations that addressed research objectives. An overview of the research questions, main themes and subthemes can be found in Table 2.

**TABLE 2:** Research questions, main themes and subthemes.

Research questions	Themes	Subthemes
Older adults’ experiences of internalized ageism in relation to digital technology use	Stereotypes and self-perceptions	– Stereotypes about older and younger digital technology users – Underestimating oneself, lack of confidence in digital skills
	Emotions and behaviors caused by ageist self-views	– Fear, anxiety and stress – Avoidance and disengagement from digital technologies
Older adults’ experiences of relational ageism in relation to digital technology use	Ageism in everyday interactions concerning digital technology use	– Covert/subtle ageism: help, protection, humor, respect or attention – Overt ageism: age-related bias, frustration and impatience
	The interplay of relational and internalized ageism in digital technology use	– Relational ageism triggering and facilitating identification with stereotypes
Confident and hesitant technology users’ experiences of ageism	Paradoxical impact of experiences of ageism – ageism hinders or motivates digital technology use	– Confident users: internalized ageism leads to greater self-compassion, efforts to adapt and compensate – Confident users: confronting or dismissing stereotypes in reaction to relational ageism – Hesitant users: avoidance and disengagement from technologies in response to ageism
	Interactions that either reinforce or challenge ageist stereotypes	– Confident users: others praising and encouraging independent use of technologies, offering help if needed – Hesitant users: others taking over or encouraging to stop using digital technologies

## 3 Results

### 3.1 Older adults’ experiences of internalized ageism in relation to digital technology use

When analyzing how internalized ageism was reflected in participants’ narratives regarding digital technology use, two major themes emerged. The first encompassed various stereotypes and self-perceptions expressed by the participants. The second reflected the emotional and behavioral consequences of ageist self-views. We will further examine these themes and their related subthemes.

#### 3.1.1 Stereotypes and self-perceptions

More than half of the participants expressed various stereotypes about both older and younger digital technology users. These stereotypes mostly concerned the belief that there is an age limit for learning or successfully using digital technologies. Tom explained this “age limit” through social comparisons between younger and older technology users − a pattern that appeared frequently in participants’ narratives:

*Because for them* (young people) *work and skills were about that* (digital technology use), *but an older person, if they didn’t need to deal with that before, well, what will they learn now?* (Tom)

An additional subtheme involved participants underestimating themselves as digital technology users and lacking confidence in their skills and abilities. Mary reflected on how she viewed herself as a digital technology user and how her age impacted her confidence:

*Well to say it like that, I’m chasing this passing train in all questions no matter what I’m doing. < … > . Yes, this (*age*) also affects me, this confidence in myself.* (Mary)

#### 3.1.2 Emotions and behaviors caused by ageist self-views

Another theme captured the emotions and behaviors related to digital technology use that participants associated with their age. Participants who expressed or identified with age stereotypes commonly reported feeling fear, anxiety, and stress when using or anticipating the use of digital technologies. They feared making mistakes, causing problems, and encountering unsafe situations when using such technologies. For Valerie, using digital technologies caused stress, partly due to stereotypical assumptions about her inability to handle challenges if they occurred:

*It’s already too difficult for me. < … > . A young person might still understand what went wrong, but someone older often can’t even realize that something was done incorrectly*. (Valerie)

Related subtheme concerned avoidance and disengagement from digital technologies. Participants mostly associated this avoidance with negative past experiences using technologies, aging features, or health problems. However, in some cases, we noticed that these experiences triggered age salience, making participants more aware of their age, and facilitated identification with ageist stereotypes. Thus, this avoidance could be seen as a strategy to prevent fulfilling negative expectations mentioned earlier (e.g., making mistakes). For example, Elena shared that arthritis had caused her to lose confidence in her ability to successfully use e-banking services:

*I used to know how to do it* (use e-banking services), *but now, arthritis, my fingers are numb. < … > . You can’t move them fast, and you’re running out of time. < … > . I said, I don’t need it anymore, I won’t do it anymore, it’s for those who can, for those who are not so old, healthy.* (Elena)

Although illness had a direct impact on her use of e-banking services, it also reinforced her belief that it was too complicated for her due to her age and health condition. This is why her avoidance could be seen as caused by ageist self-views.

### 3.2 Older adults’ experiences of relational ageism in relation to digital technology use

When analyzing older adults’ experiences of relational ageism in relation to digital technology use, two major themes became prominent. The first encompassed ageism in everyday interactions concerning digital technology use, while the second reflected the interplay between internalized and relational ageism regarding participants’ use of digital technologies.

#### 3.2.1 Ageism in everyday interactions concerning digital technology use

When asked directly, most participants stated that they didn’t experience relational ageism. However, covert and overt forms of relational ageism were noticeable in their reported everyday interactions.

About half of the participants shared experiences that involved subtle forms of relational ageism. In these experiences, ageism was often disguised as care or help (e.g., doing digital tasks for older people instead of showing them how), humor, respect, attention, or protection from digital dangers. Few participants noted receiving exaggerated attention or respect as they aged, feeling “watched over” by others, which affected their confidence with digital technologies. Evelyn’s experience in her workplace illustrates how this type of attention could trigger age salience, as she felt singled out because of her age:

*I started to notice this exaggerated attention, that somehow, they (her colleagues) are talking with much respect. < … > . It wasn’t anything bad. < … > . I didn’t want this exaggerated attention. I understood that it is because of age. This respect is because of age. (Evelyn)*

There were also accounts of interactions containing overt relational ageism, where others more directly expressed their age-related biases (e.g., presumptions about participants’ technological competencies) or behaved in a more overtly disrespectful manner (e.g., lacking patience or showing irritation). Generational tension often occurred in their reported interactions with younger people, causing some participants to feel that asking younger relatives for help with digital technologies was unhelpful. Natalie likely felt misunderstood and undervalued, as her discomfort with smartphones was blamed on incompetence rather than design features that were inconvenient for her:

*For me, it’s badly designed, it’s not convenient. And my granddaughter* (says)*: “That’s because you don’t know how to* (use it)*, grandma.” Here you go. I mean, I know how to do a lot of things, but here, “You don’t know how to.”* (Natalie)

#### 3.2.2 The interplay of relational and internalized ageism in digital technology use

Experiences of relational ageism, both covert and overt, seemed to shape digital technology use in similar ways to internalized ageism - participants expressing a lack of confidence in their skills and abilities, fearing or avoiding technology. Thus, the theme of the interaction between relational and internalized ageism emerged from participants’ narratives, with relational ageism potentially reinforcing internalized ageism in digital technology use. In some cases, experiences of relational ageism made participants more aware of their age and triggered ageist stereotypes, which they then applied to themselves, disrupting their use. As mentioned earlier, Evelyn didn’t like the “exaggerated attention or respect” she received at her workplace, which she associated with her age. This experience made her doubt her ability to complete digital tasks successfully, which eventually influenced her decision to retire early to avoid fulfilling negative expectations:

*And then you have to keep up, maintain a certain level. Maybe I will forget, maybe I won’t say something right. Especially because I was very worried about digital technologies. I was very scared to fill in all the documents on the computer. It was already too difficult for me.* (Evelyn)

### 3.3 Confident and hesitant technology users’ experiences of ageism

#### 3.3.1 Paradoxical impact of experiences of ageism–ageism hinders or motivates digital technology use

When analyzing the experiences of ageism among confident and hesitant digital technology users, the paradoxical impact of ageism emerged. Only confident users responded to ageism in ways that contradicted common stereotypes and typical reactions, revealing its paradoxical effect. 3 out of 6 confident participants shared such experiences. For these individuals, ageism appeared to be a motivating factor for using digital technologies, which was the most unexpected finding of the study.

Laura, for example, associated her technology-related difficulties with her age, demonstrating internalized ageism. However, instead of discouraging her, this perspective led her to feel more compassion for herself. She saw the technology-related problems not as personal failings but as age-related challenges, which helped to reassure her and fostered self-acceptance. This, in turn, motivated her to find ways to adapt and minimize difficulties, rather than to avoid or give up on using technology. In this unusual way, the activation and identification with age-related stereotypes actually fostered greater self-compassion and supported digital technology use:

*If you do something and it doesn’t work out, well yes, you’re older, it’s understandable, calm down. < … > . You need to do it many times. < … > . I even write down what others tell me. < … > . Because I know I can’t trust myself, but I need this external… well not help, but kind of help for myself, so that I can remember better. (Laura)*

Confident digital technology users were also able to reject stereotypes and maintained their use despite others’ stereotypical views. Experiences of relational ageism seemed to motivate them to use digital technologies, either as a way to improve in areas targeted by age stereotypes or to challenge those stereotypes. This was evident in Monica’s remarks:

*I don’t understand how you can, well, accept all that nonsense. Who cares who says what. But at the same time it’s a way of seeing where you must try harder, to push yourself, to not give up*. (Monica)

Mary also shared a similar sentiment in challenging the stereotypical assumptions she reported from others:

*To me it’s like this, some people say that: “At his age you don’t really need anything anymore.” But I need everything. I don’t say: “Oh, how much time do I have left, it doesn’t matter.” No - I still need everything. I say, as long as a person is alive, they need things. This is why I look at it (*digital technology use*) optimistically.* (Mary)

In contrast, among hesitant digital technology users, the experiences of ageism seemed to disrupt their use. Only hesitant users exhibited avoidance and withdrawal from digital technologies in response to ageism. This withdrawal was often accompanied by reliance on others to do digital tasks for them. For example, Evelyn avoided learning opportunities and instead relied on customer service workers to help her extend her insurance policy via the app, due to negative expectations of herself:

*They (*customer service staff*) are like: “You can come, if you can walk, we will show you.” And I say: “What if I come and pay but I won’t look at what you’re showing me? < … > . I myself don’t have a wish to do this with technology, because later, I think like that, I will get burdened with everything, and then everything will be gone, and I will be left alone.* (Evelyn)

#### 3.3.2 Interactions that either reinforce or challenge ageist stereotypes

In the confident participants’ shared experiences, the role of positive and empowering interactions emerged. In these experiences, other people acknowledged or praised their digital technology skills and efforts. A sense of pride was evident when Laura shared how her daughter perceived her as a digital technology user:

*And when I ordered there for the first time on online shop. < … > . She* (the daughter) *said: “Well, you know everything already (laughs)”… yes, yes she says: “What more can I teach you?”* (Laura)

Additionally, only confident users described interactions in which others encouraged them to use digital technologies independently − offering help only when genuinely needed, rather than completing tasks for them. Violet’s interaction with her daughter illustrates such empowering support, which was reflected in her own proactive attitude and confidence in her ability to learn:

*I say: “Now I don’t have time, you do it, you’ll do it faster.” < … >* (the daughter): *“No, try it yourself.” < … > . When I really need it she does it for me, but still, the more you do it yourself, the more you learn something. (Violet)*

Only hesitant users shared experiences when others took over their digital technology use or encouraged them to stop using technologies. These experiences tended to confirm ageist stereotypes and disrupt their use. For example, Elena’s husband discouraged her from buying a smartphone by assuming she wouldn’t have any use for it. Unfortunately, this reinforced her belief that smartphones are not relevant to her, and that not using them helps avoid causing problems:

*It seems to me that he* (the husband) *understands that I’m not going to go there on the internet, I won’t be using all of these functions, why do I need it? I might lose it, make some mistakes, somebody will scam me.* (Elena)

## 4 Discussion

This study showed that, for older digital technology users, ageism was an important factor of their use of digital technologies and should be incorporated into future research exploring the causes of, and solutions to, the digital divide among older adults. This conclusion is based on several key findings from the study, which we will discuss further.

First, the study revealed the potential multifaceted impact of internalized ageism, which related to participants’ use of digital technologies through attitudinal, emotional, and behavioral pathways. Participants expressed stereotypical views about both younger and older technology users. When identifying with ageist stereotypes, they lacked confidence in their digital abilities, experienced fear, anxiety, and stress when using technologies, and avoided or withdrew from them. These factors have been replicated in other studies as consequences of internalized ageism (Schreurs et al., 2017; Barrie et al., 2021; Köttl et al., 2021b). Such a multifaceted impact of internalized ageism can be explained using the Risk of Ageism model (Swift et al., 2017), which suggests that the internalization of stereotypes can lead to ageist attitudes and deficit-based self-views. The second pathway, stereotype threat, could explain how ageism leads to anxiety and stress when interacting with digital technologies. This, in turn, can cause avoidance and withdrawal to prevent confirming negative expectations, as acknowledged by other research (Levy, 2009; Ivan and Cutler, 2021). Despite the growing recognition of the need to explore the multifaceted impact of ageism, there is a lack of studies that have empirically documented it (Levy and Apriceno, 2019; Chang et al., 2020). Thus, the present study contributes to the field by indicating the potential multifaceted impact of internalized ageism on digital technology use and emphasizing the need for further research on this topic.

Participants also described experiencing age-related bias, frustration, and impatience from others in relation to their use of digital technologies. These findings are based on their experiences and perceptions of their social environment and thus reflect subjective interpretations. However, similar findings have been observed in other studies (McCausland et al., 2015; Vaportzis et al., 2017). The study also revealed that relational ageism can manifest in subtle ways in everyday interactions involving digital technology use − such as humor, attention, help, or protection − that participants may not recognize as ageist. This aligns with other research showing that subtle forms of ageism are common and often unrecognized as discriminatory (Schreurs et al., 2017; Köttl et al., 2021b), reflecting a societal tendency to rarely question ageist behaviors unless they are overtly harmful (Chen et al., 2018; Stubbe, 2021). Yet, research indicates that even benevolent ageism or positive aging stereotypes can significantly impact older adults’ emotional states or self-perceptions (Cuddy and Fiske, 2002; Miguel and Carvalhais, 2025). In this study, these subtle experiences of relational ageism appeared to relate to digital technology use in a similar way to overt forms − undermining participants’ technological confidence, increasing anxiety and avoidance. This underscores the need to further study and include both overt and subtle forms of relational ageism in future research.

The study also uncovered a possible relationship between internalized and relational ageism experiences, with the latter reinforcing the former, suggesting a potential mechanism through which ageism manifests and influences older adults’ use of digital technologies. Experiences of relational ageism appeared to trigger age salience, made older adults more aware of their age and age stereotypes, and facilitated identification with them, which disrupted digital technology use. Some researchers consider ageism to be an interpersonal stressor, while internalized ageism is viewed as internalized microaggressions within relationships (Gendron et al., 2018, 2020). Previous research has demonstrated that ageism operates through a combination of internal and external factors (Choi et al., 2021; Köttl et al., 2021b), yet the mechanisms through which these factors interact remain insufficiently explored. Some scholars argue that as long as individuals do not internalize stereotypes, relationships may not affect their self-perception (Minichiello et al., 2000), while others suggest that people don’t need to agree with the stereotypes for them to influence behavior and performance (Lamont et al., 2015). Stereotype threat is described as situational and fluid − it needs to be activated to exert its influence (Steele and Aronson, 1995). In this study, it can be speculated that relational ageism experiences functioned as such situational triggers, making participants feel that they were perceived differently due to their age. This, in turn, could have reinforced internalized ageism, as in activated stereotypes, and facilitated identification with them, thereby shaping how participants felt and engaged with technology. Prior research has emphasized the importance of environments that do not reinforce ageist stereotypes (Barrie et al., 2021). Accordingly, the findings of this study contribute to the field by highlighting the interaction between older adults’ internal and external experiences of ageism and suggesting a potential mechanism through which this interaction may shape their digital technology use.

The results also showed that experiences of ageism differed among confident and hesitant users, providing insight into how these groups may experience and react differently to ageism. Regarding the external environment, confident users appeared to benefit from people around them who provided positive feedback about their digital technology use and efforts. They also shared interactions where others created opportunities to empower them − encouraging independent use of digital technologies while offering help if needed. Hesitant users, on the other hand, reported interactions where others took over their digital technology use, or encouraged them to stop using such technologies. Such diverse experiences seemed to impact participants’ self-views and behavior − either reinforcing or challenging stereotypical self-beliefs and shaping how they interact with technologies. Although the relational ageism experiences in this study reflect participants’ perceptions rather than objective observations of their social environments, environmental support has been shown to be central to older adults’ adoption of digital technology, while disempowering interactions exacerbate technological challenges (Schreurs et al., 2017; Köttl et al., 2021b; McCashin et al., 2022). The current findings provide a closer look at how helpful versus unhelpful support in digital technology use may impact older adults through reinforcing or challenging ageist stereotypes. Additionally, mastering technologies has been shown to help reinforce positive self-views or counter ageist beliefs (Köttl et al., 2021a,b; Ivan and Cutler, 2021). Thus, confident participants may have benefited from interactions that helped them learn and confront stereotypes, with mastery and use of technology further challenging those stereotypes. In contrast, hesitant users could have experienced the opposite.

The most unexpected finding of this study was the paradoxical impact of ageism − where experiences of ageism could either hinder or motivate digital technology use among confident and hesitant users. Although the small sample limits this finding to an exploratory insight rather than a definitive conclusion, its novelty provides valuable theoretical implications, warrants further investigation, and justifies discussion. In our study, only hesitant users appeared to disengage from or avoid using digital technologies in response to their own or others’ limiting views of aging. This aligns with previous studies showing that internalized ageism can lead to avoidance, withdrawal, or skepticism toward technology (Vaportzis et al., 2017; Köttl et al., 2023; Mannheim et al., 2023b). The positive, motivating impact of ageism appeared only among confident users. For them, the experiences of ageism seemed to motivate digital technology use − either as a way to confront stereotypes or to approach technological challenges with greater self-compassion. Previous research has shown that some individuals cope with prejudice by employing adjustment strategies, such as behaving in ways that disconfirm stereotypes or acting in more socially skilled manners (Choi et al., 2021; Farmosa, 2021). However, the finding that internalized ageism can foster self-compassion and motivate the use of digital technology is, to our knowledge, novel. Few studies have explored how reactions to internalized ageism, such as disengagement from technologies or attributing technology-related problems to one’s age, might function as a defense mechanism to preserve a positive self-identity (van der Horst, 2019; Köttl et al., 2021b). However, such processes tend to reduce digital engagement. In contrast, this study suggests that attributing technology related issues to age may help preserve positive self-identity while supporting technology use and confidence. This indicates that the impact of ageism is not universal or predictable. The finding that only confident technology users encountered this paradoxical impact of ageism allows us to speculate they may possess resilience factors, which could stem from their broader environment, interactions with others or personal traits − areas that warrant further investigation. In addition to efforts to reduce ageism in society, it is also important to equip older adults with the skills and knowledge necessary to manage ageism in more adaptive and constructive ways.

It is also important to consider the socio-economic context in which this study was conducted. Lithuania’s socio-cultural factors could have shaped how older adults experienced ageism in relation to digital technology use. Although the Baltic states have invested in digital literacy, significant skill gaps remain among older generations due to the late onset of digitalization after the collapse of the Soviet Union (Vyriausybës strateginës analizës centras, 2020). Unlike in other European regions, e.g., in the Nordic countries, where active aging policies and observed positive attitudes of aging could support digital inclusion (Kalfoss, 2017; Aidukaite and Ainsaar, 2022), attitudes toward aging in Lithuania and other former socialist states tend to be less positive (Rychtaøíková, 2019). Lithuanian cultural traditions have historically emphasized respect for elders and the importance of family support (Sundstrom et al., 2007), however, these norms are shifting due to changes in family dynamics and pressures on healthcare and welfare resources (Eurostat, 2015; Vârpin, 2018). While digital inclusion initiatives are expanding (Stumbrys et al., 2022), inequalities in access persist, particularly when compared with wealthier European countries such as the Nordic states (Hilmarsson, 2021). These historical, cultural, and economic dynamics could influence both digital engagement and experiences of ageism among older Lithuanians, and should be considered when interpreting the findings of this study. At the same time, the results point to the importance of exploring how similar patterns may manifest across different socio-cultural contexts.

In conclusion, this study demonstrated that for older Lithuanian digital technology users ageism was an important factor related to their use of digital technologies. While internalized ageism appeared to have a multifaceted impact on digital technology use (shaping beliefs, emotions, and behaviors), relational ageism triggered ageist stereotypes and facilitated identification with them. Relational ageism experiences manifested in both subtle and overt ways. However, both forms were similarly associated with negative patterns of digital technology use. Confident and hesitant digital technology users had distinct experiences and reactions to ageism. Confident users seemed to benefit from interactions that helped them resist ageist stereotypes, while hesitant users’ interactions tended to reinforce those stereotypes. For confident users, ageism appeared to motivate digital technology use − either through self-compassion or a desire for improvement − whereas for hesitant users, it appeared to hinder use through avoidance and withdrawal. The paradoxical impact of ageism − where ageism experiences may either hinder or motivate technology use − highlights that how individuals interpret and respond to ageism can be as important as the experience itself. This underscores the need for further research into this paradoxical impact of ageism and into individual differences to better understand why some people might be more vulnerable, while others more resilient to its effects. Overall, the findings of this study emphasize the importance to integrate ageism into future research to better understand both the causes of and potential solutions to the digital divide among older adults.

### 4.1 Study limitations and practical implications

This study has several limitations that should be considered when interpreting the findings. The sample was small (13 participants), and while thematic saturation was reached in this dataset, additional participants could have introduced new perspectives, limiting the generalizability of the findings. Focus groups were “mostly confident” and “mostly hesitant” rather than strictly divided by digital technology user type, which may have influenced group dynamics. Participants were recruited mainly from senior activity centers and social media, which underrepresents less socially or digitally engaged older adults who may experience more ageism, as shown in previous studies (Chang et al., 2020; Köttl et al., 2021b; van Kampen et al., 2023). Future studies could broaden recruitment by partnering with community organizations, ongoing research projects, or using methods such as direct mailing to reach these groups (Nkimbeng et al., 2020; Köttl et al., 2021b).

In addition, sociodemographic factors such as gender, education, and socioeconomic status were not examined, although they can influence both digital technology use and experiences of ageism. For example, ageism among older women may be more strongly associated with lower internet use than among men (Choi et al., 2020), and lower education or socioeconomic status may reinforce both digital exclusion and ageism (Rippon, 2016; Chang et al., 2020; Sun et al., 2020). Similarly, user group classifications were based solely on self-rated skills and frequency of use, without capturing other dimensions of confidence such as personal relevance, purpose of use, or social support (Leone et al., 2018; Juuti et al., 2022; Baek, 2025).

Thematic analysis was primarily conducted by the first author, which may introduce bias, although coding was refined collaboratively within the research team. Finally, the cross-sectional qualitative design limits causal inference, and findings reflect the Lithuanian context. A larger and more diverse sample, along with stronger methodological rigor and the inclusion of additional relevant variables, could reveal further patterns and variations in experiences, particularly given that this topic is relatively new and underexplored.

Despite these limitations, the results of this study could provide important information for practical implications, particularly when replicated in other socio-cultural contexts. Digital literacy programs could address the potential impact of internalized ageism, helping participants build confidence and positive self-perceptions around technology use. Ageism-awareness campaigns and peer support initiatives could reduce relational ageism in digital learning environments, creating interactions that empower rather than reinforce negative stereotypes. The results also provide information about potential benefit of digital skills building programs to be personalized and differentiate between different user types. Future digital literacy interventions should address ageism and be guided by further research on its influence on digital technology use, as well as on individual differences in resilience or vulnerability to ageism.

## Data Availability

The datasets presented in this article are not readily available due to ethical considerations and participant confidentiality. Only anonymized excerpts are included in the manuscript. Requests to access the datasets should be directed to gabriele.gudynaite@fsf.stud.vu.lt.
